# Ursonic acid from medicinal herbs inhibits PRRSV replication through activation of the innate immune response by targeting the phosphatase PTPN1

**DOI:** 10.1186/s13567-024-01316-8

**Published:** 2024-05-23

**Authors:** Yuanqi Yang, Yanni Gao, Haifeng Sun, Juan Bai, Jie Zhang, Lujie Zhang, Xing Liu, Yangyang Sun, Ping Jiang

**Affiliations:** 1 Key Laboratory of Animal Disease Diagnostics and Immunology, Ministry of Agriculture, MOE International Joint Collaborative Research Laboratory for Animal Health & Food Safety, College of Veterinary Medicine, Nanjing Agricultural University, Nanjing, 210095 China; 2 Jiangsu Co-Innovation Center for the Prevention and Control of Important Animal Infectious Diseases and Zoonoses, Yangzhou University, Yangzhou, 225009 China

**Keywords:** Ursonic acid (UNA), PRRSV, protein tyrosine phosphatase nonreceptor type 1 (PTPN1), antivirals

## Abstract

Porcine reproductive and respiratory syndrome (PRRS), caused by the PRRS virus (PRRSV), has caused substantial economic losses to the global swine industry due to the lack of effective commercial vaccines and drugs. There is an urgent need to develop alternative strategies for PRRS prevention and control, such as antiviral drugs. In this study, we identified ursonic acid (UNA), a natural pentacyclic triterpenoid from medicinal herbs, as a novel drug with anti-PRRSV activity in vitro. Mechanistically, a time-of-addition assay revealed that UNA inhibited PRRSV replication when it was added before, at the same time as, and after PRRSV infection was induced. Compound target prediction and molecular docking analysis suggested that UNA interacts with the active pocket of PTPN1, which was further confirmed by a target protein interference assay and phosphatase activity assay. Furthermore, UNA inhibited PRRSV replication by targeting PTPN1, which inhibited IFN-β production. In addition, UNA displayed antiviral activity against porcine epidemic diarrhoea virus (PEDV) and Seneca virus A (SVA) replication in vitro. These findings will be helpful for developing novel prophylactic and therapeutic agents against PRRS and other swine virus infections.

## Introduction

Porcine reproductive and respiratory syndrome (PRRS), caused by the PRRS virus (PRRSV), is a highly contagious infectious disease. It causes reproductive disorders in sows and respiratory symptoms and high mortality rates in pigs of all ages (especially piglets), leading to critical economic losses in the global swine industry [1–4]. PRRSV is an enveloped, single-stranded positive-sense RNA virus belonging to the genus *Porartevirus*, family *Arteriviridae*, and order *Nidovirales* and can be divided into type 1 and type 2 [5–7]. Although PRRS has been reported for more than 35 years, no drugs or vaccines can effectively prevent its spread. Therefore, there is an urgent need to develop new alternative strategies for preventing and controlling PRRS.

Innate immunity is the first line of defence that limits viral spread and regulates adaptive immune responses. Viral pathogen-associated molecular patterns (PAMPs) are recognized by host pathogen recognition receptors (PRRs) and subsequently trigger associated signalling pathways, which are mediated by retinoic acid-inducible gene-I (RIG-I)-like receptors (RLRs) and Toll-like receptors (TLRs). Ultimately, these intracellular signalling events lead to the production of interferons (IFNs) and proinflammatory cytokines and the activation of other downstream signalling effectors [8, 9]. During PRRSV infection, RIG-I/MDA5 recognizes dsRNA and activates type I IFN production to exert antiviral effects [10, 11]. Correspondingly, PRRSV has evolved numerous strategies to evade type I IFN restriction. For example, some proteins of PRRSV, including nsp1α, nsp1β, nsp2, nsp4, nsp7, nsp11, and N, have been shown to downregulate IFN production [12–18].

Protein tyrosine phosphatase nonreceptor type 1 (PTPN1), an essential protein tyrosine phosphatase (PTP), plays a vital role in various cellular processes and is involved in the infection of multiple viruses, such as hepatitis C viruses, baculovirus, dengue virus, and HSV-1 [19–23]. PTPN1 has been reported to dephosphorylate STING at Y245, leading to STING degradation via the ubiquitin-independent 20S proteasomal pathway and suppressing the innate immune response to DNA viruses [24]. Recently, Compound-182 and ABBV-CLS-484, which are highly potent and selective active site competitive inhibitors of PTPN1 and PTPN2, respectively, were reported to enhance T-cell antitumour immunity [25, 26]. However, the impact of PTPN1 on antiviral innate immunity against RNA viruses is still unclear.

Ursonic acid (UNA) is a natural pentacyclic triterpenoid extracted from a great variety of traditional medicinal herbs, such as *Ziziphus jujuba*, *Crataegus pinnatifida*, and *Malus baccata*. UNA has many biological and pharmacological properties, including anticancer, antiprotozoan, anti-inflammatory, and antiviral activities [27]. Early in 1987, Poehland et al. reported that UNA suppressed the cytopathic effects of HSV-1 and HSV-2 in Vero cells [28]. In recent years, the antiviral activities of UNA against HIV-1 and SARS-CoV-2 have also been reported [29, 30]. However, the underlying mechanism of UNA antiviral activity is still unknown.

Here, we showed that the natural product UNA significantly inhibited PRRSV infection at micromolar concentrations in a dose-dependent manner in Marc-145 cells and PAMs. Protein interference and phosphatase activity assays revealed that UNA targeted PTPN1 and suppressed its phosphatase activity. PTPN1 suppressed RLR-mediated IFN-β production and promoted PRRSV infection, both of which were dependent on its phosphatase activity. Further study proved that the inhibition of PRRSV and the promotion of IFN-β production by UNA both depended on PTPN1. We also found that UNA had a relatively broad-spectrum antiviral effect on RNA viruses such as porcine epidemic diarrhoea virus (PEDV) and Seneca virus A (SVA). Overall, this study is the first to reveal the anti-PPRSV activity of UNA and the underlying novel mechanisms involved. PTPN1, a negative regulator of the RLR-mediated antiviral signalling pathway, could be a potential antiviral target for drug design.

## Materials and methods

### Cells, viruses, and reagents

Marc-145 cells (ATCC CRL-12231), Vero cells (ATCC CCL-81), ST cells (ATCC CRL-1746), and HEK-293 T cells (ATCC CRL-3216) were cultured at 37 °C with 5% CO_2_ in Dulbecco’s modified Eagle’s medium (DMEM; Invitrogen, USA) supplemented with 10% foetal bovine serum (FBS; Gibco, USA) and penicillin (250 U/mL)-streptomycin (250 μg/mL). Porcine alveolar macrophages (PAMs) were collected from the lung lavages of 4 week-old piglets (free of PRRSV, PCV2, and PRV infections) as previously described [31] and cultured in Roswell Park Memorial Institute 1640 medium (RPMI 1640; Gibco, USA) supplemented with 10% FBS and penicillin (250 U/mL)-streptomycin (250 μg/mL) at 37 °C with 5% CO_2_.

The highly pathogenic PRRSV strain BB0907 (GenBank accession no. HQ315835.1) was used for all experiments and is referred to as “PRRSV” throughout this article. The other two PRRSV-2 strains, S1 (a classical strain; GenBank accession no. DQ459471.1) and FJ1402 (an NADC30-like strain; GenBank accession no. KX169191.1), were used and are specifically mentioned by their names. Porcine epidemic diarrhoea virus (PEDV) YZ (GenBank accession no. MK841495.1), which was passaged in Vero cells, and Senecavirus A (SVA) CH-SD (GenBank accession no. MH779611.1), which was passaged in ST cells, were both maintained in our laboratory.

The anti-PRRSV N protein, anti-SVA VP2 protein, and anti-PEDV N protein were all prepared in our laboratory. Anti-HA, anti-GAPDH, anti-PTPN1, and Alexa Fluor 488-conjugated goat anti-mouse IgG (H + L) were obtained from Proteintech (USA). The anti-His antibody was obtained from Yifeixue (China), and the anti-myc antibody was obtained from Abmart (China). Horseradish peroxidase (HRP)-conjugated goat anti-rabbit and anti-mouse IgG (H–L) secondary antibodies were obtained from Beyotime (China). Ursonic acid (UNA, purity ≥ 99.82%), sodium orthovanadate (purity ≥ 99.88%), and pNPP (purity ≥ 99.93%) were purchased from Selleck Chemicals (USA). 4-Propanesulfonyl morpholine (MOPS, purity ≥ 99%), sodium acetate (purity ≥ 99.9%), ethylenediaminetetraacetic acid (EDTA, purity ≥ 99.5%), and sodium chloride (purity ≥ 99.9%) were purchased from Solarbio (China).

### Cytotoxicity assay

The test compounds were added to the corresponding cells and incubated for 24–48 h at 37 °C. Cell viability was tested using an enhanced Cell Counting Kit-8 (CCK-8; Beyotime, China) following the manufacturer’s instructions. An equal volume of DMSO was used as the control. The 50% cytotoxic concentration (CC_50_) was calculated using GraphPad Prism 7.0 software.

### Antiviral activity assay

To investigate the antiviral effects of UNA on PRRSV (0.1 or 1 MOI), PEDV (0.1 MOI), and SVA (0.02 MOI), Marc-145 cells, PAMs, Vero cells, and ST cells were infected with the indicated viruses. After incubation at 37 °C for 1 h, the supernatants were removed, and DMEM supplemented with 2% FBS containing different concentrations of UNA or an equal volume of DMSO was added. Finally, cells and supernatants were collected at the indicated time points, and the virus titre, number of virus-infected cells, viral protein levels, and viral RNA levels were evaluated by endpoint dilution assay, IFA, Western blot analysis, and RT‒qPCR, respectively. The fluorescence intensity was determined by ImageJ software, and the 50% effective concentration (EC_50_) was estimated by GraphPad Prism 7.0 software.

### Western blot analysis

Cells were lysed with radioimmunoprecipitation assay (RIPA) lysis buffer (Beyotime, China) on ice for 15 min, separated by SDS‒PAGE and transferred to a nitrocellulose membrane. The membrane was then incubated in blocking buffer (5% nonfat milk in PBST, w/v) for 2 h at room temperature (RT), washed with PBST and then probed with the corresponding primary antibodies for 2 h at RT. After incubation with the corresponding secondary antibodies for 1 h and treatment with an enhanced chemiluminescence (ECL) kit (Tanon, China), the specific bands were analysed using a Tanon 5200 chemiluminescence imaging system (Tanon, China). The bands were quantified using ImageJ (v. 5.1) and normalized to the levels of GAPDH.

### Quantitative reverse transcription PCR (RT‒qPCR)

Total RNA was extracted from the cells using a Total RNA Kit I (Omega Bio-Tek, USA). cDNAs were then synthesized using HiScript qRT SuperMix (Vazyme, China) following the manufacturer’s instructions. RT‒qPCR was performed by an ABI QuantStudio 6 System (Applied Biosystems, USA) using AceQ^®^ qPCR SYBR^®^ Green Master Mix (Vazyme, China). The data are presented as the fold change in gene expression normalized to that of the housekeeping gene and relative to that of the mock-infected control. Each reaction was performed in triplicate. The relative gene expression levels were calculated through the 2^−ΔΔCt^ method, and the results were calculated as the mean ± standard deviation (SD). The primers used for qPCR are listed in Table 1.

**Table 1:** **Primers used for qPCR analysis**

Primer name	Sequence (5ʹ—3ʹ)
PRRSV ORF7-F	AAACCAGTCCAGAGGCAAG
PRRSV ORF7-R	TCAGTCGCAAGAGGGAAAT
PEDV N-F	TTCTTGTTTCACAGGTGGATG
PEDV N-R	GCTGCTGCGTGGTTTCA
SVA VP2-F	CTCCACCTCGGTAGACATA
SVA VP2-R	GGGACAAGCACCATAACA
chloPTPN1-F	GCTTCTCCTACCTGGCTGTG
chloPTPN1-R	CTCATCCTTCACCCACTGGT
susPTPN1-F	GCTCAACAGAGTGATGGA
susPTPN1-R	CGGACGGTGTAATATGACT
chloIFN-β-F	GCTGGAATGAGACTATTGTG
chloIFN-β-R	CCTTCAGGTAATGCAGAATC
chloISG15-F	CAAAGATCGCCCAGAAGA
chloISG15-R	GGATGCTCAGAGGTTCAT
chloISG56-F	TCATCAGGTCAAGGATAGTC
chloISG56-R	CATAGGCTAGTAGGTTGTGT
chloGAPDH-F	CCTTCCGTGTCCCTACTGCCAA
chloGAPDH-R	GACGCCTGCTTCACCACCTTCT
susβ-actin-F	CTCCATCATGAAGTGCGACGT
susβ-actin-R	GTGATCTCCTTCTGCATCCTGTC

chlo refers to *Chlorocebus sabaeus*, and sus refers to *Sus scrofa.*

### Immunofluorescence assay (IFA)

Marc-145 cells grown in 48- or 96-well plates were treated as indicated. Post-treatment, the cells were rinsed twice with PBS and fixed with 4% paraformaldehyde (PFA) for 10 min. The cells were subsequently permeabilized with 0.2% Triton X-100 for 10 min and blocked with 2% bovine serum albumin (BSA) in PBS for 1 h. Following incubation with the primary antibody at 4 °C overnight, the cells were washed and incubated with the Alexa Fluor 488-conjugated secondary antibody (green) for 1 h. The nuclei were stained with 4,6-diamidino-2-phenylindole (DAPI; Beyotime, China) (blue) for 10 min. After washing with PBST, immunofluorescence was observed using a Zeiss inverted fluorescence microscope.

### Virus titration

Cells grown in 96-well plates were infected with tenfold serial dilutions of virus samples. After 1 h at 37 °C, the culture medium was replaced with fresh DMEM supplemented with 2% FBS. Viral titres were determined at the corresponding times (2–5 days post-inoculation (dpi)) using endpoint dilution analysis. The Reed-Muench method was used to calculate the median tissue culture infectious dose (TCID_50_).

### Virucidal activity assay

To determine whether UNA directly inactivates PRRSV, DMSO or UNA (10 µM) was incubated with PRRSV (0.1 or 1 MOI) at 37 °C for 3 h. The mixtures were subjected to virus titration.

### Time-of-addition assay

To evaluate which stages of the PRRSV life cycle are affected by UNA, a time-of-addition assay was performed, as shown in Figure 2A. Marc-145 cells seeded into 24-well plates were either pre, co, or post-treated with UNA relative to PRRSV infection. The experiment began when the cells reached 80% confluence and was noted as − 1 h. At − 1 h, the cells in the pre-treated group were treated with UNA (10 µM) for 1 h. The culture supernatants were discarded, and DMEM + PRRSV (0.1 MOI) was added for another hour, followed by the addition of DMEM supplemented with 2% FBS. At 0 h, the cotreated group was treated with UNA (10 µM) + PRRSV (0.1 MOI), and the post-treated group was infected with PRRSV (0.1 MOI). At + 1 h, the culture supernatants in the post- or cotreated groups were replaced with DMEM-2% FBS with or without UNA (10 µM), respectively. The infection of all groups continued for 48 h.

### Plasmid construction

Total RNA was extracted from Marc-145 cells, and cDNAs were then synthesized as previously described. *Chlorocebus sabaeus* PTPN1 (chloPTPN1) was generated by PCR amplification of cDNA. The sequence of the amplification product was restriction digested and cloned and inserted into pCAGGS (vector) with an HA or myc tag at its 3ʹ end to produce pCAGGS-chloPTPN1-HA. Next, the enzyme activity mutant plasmids of PTPN1 were obtained by overlap PCR based on pCAGGS-chloPTPN1-HA. In addition, chloPTPN1 was cloned and inserted into pET-28a for prokaryotic expression of PTPN1. The primers used for PCR are listed in Table 2.

**Table 2:** **Primers used for plasmid construction**

Primer name	Sequence (5ʹ—3ʹ)
chloPTPN1-HA-F_*Eco*RI	CCGAATTCATGGAGATGGAAAAGGAG
chloPTPN1-HA-R_*Kpn*I	CGGGTACCCTAAGCGTAATCTGGAACATCGTATGGGTATGTGTTGCTGTTGAACA
chloPTPN1-myc-R_*Kpn*I	CGGGTACCCTACAGATCCTCTTCTGAGATGAGTTTTTGTTCTGTGTTGCTGTTGAACA
chloPTPN1_D181A-F	TTCCACTATACCACATGGCCCGCCTTTGGAGTCCCCGAATCGCCA
chloPTPN1_D181A-R	TGGCGATTCGGGGACTCCAAAGGCGGGCCATGTGGTATAGTGGAA
chloPTPN1_C215S-F	CACGGGCCCGTCGTGGTGCACTCCAGTGCGGGTATCGGCAGGTCT
chloPTPN1_C215S-R	AGACCTGCCGATACCCGCACTGGAGTGCACCACGACGGGCCCGTG
chloPTPN1-F_*Eco*RI	CGGAATTCATGGAGATGGAAAAGGAGTTCGAGCAG
chloPTPN1-R_*Sal*I	GTGTCGACTGTGTTGCTGTTGAACAGGAACCTG

chlo refers *to Chlorocebus sabaeus*.

### Small interfering RNA assay

Marc-145 cells or PAMs were seeded in 24-well plates and transfected with 100 nM siNC, si-chloPTPN1, or si-susPTPN1 (− 1, 2, and 3; GenePharma, China) using 1.5 µL of Lipofectamine^™^ 3000 reagent (Invitrogen, USA) for 24 or 12 h. The cells were then treated with UNA and infected with PRRSV (0.4 MOI) for 36 or 24 h. The cells were harvested for Western blotting or RT‒qPCR. The sequences of the siRNAs targeting PTPN1 are listed in Table 3.

**Table 3:** **Sequences of siRNAs targeting PTPN1**

siRNA name	Sequence (5ʹ—3ʹ)
si-chloPTPN1-1-sense	CGGCCAUUUACCAGGAUAUdTdT
si-chloPTPN1-1-antisense	AUAUCCUGGUAAAUGGCCGdTdT
si-chloPTPN1-2-sense	GACCCUUCUUCCGUUGAUAdTdT
si-chloPTPN1-2-antisense	UAUCAACGGAAGAAGGGUCdTdT
si-chloPTPN1-3-sense	GAGCCACACAAUGGGAAAUdTdT
si-chloPTPN1-3-antisense	AUUUCCCAUUGUGUGGCUCdTdT
si-susPTPN1-1-sense	GGCGUUGUUAUGCUCAACAdTdT
si-susPTPN1-1-antisense	UGUUGAGCAUAACAACGCCdTdT
si-susPTPN1-2-sense	GAUCUUUGAAGACACAAAUdTdT
si-susPTPN1-2-antisense	AUUUGUGUCUUCAAAGAUCdTdT
si-susPTPN1-3-sense	CCUCAUUCCUGAACUUUCUdTdT
si-susPTPN1-3-antisense	AGAAAGUUCAGGAAUGAGGdTdT
siNC-sense	UUCUCCGAACGUGUCACGUdTdT
siNC-antisense	ACGUGACACGUUCGGAGAAdTdT

chlo refers to *Chlorocebus sabaeus*, and sus refers to *Sus scrofa.*

### Target protein prediction

SwissTargetPrediction is an online server used for predicting the most likely protein targets of small molecules by mapping the similarities between their structures and known ligands [32]. The structure of UNA from the PubChem database was input into SwissTargetPrediction for the identification of potential drug targets in *Homo sapiens* [33, 34].

### In silico docking

The crystal structure of *Homo sapiens* PTPN1 (homoPTPN1) was obtained from the Protein Data Bank (PDB: 2F71). Due to the lack of crystal structures, the amino acid sequences of chloPTPN1 and *sus scrofa* PTPN1 (susPTPN1) were obtained from UniProt A0A0D9RRG7_CHLSB and A0A8D0SH29_PIG, respectively. The 3D structures of chloPTPN1 and susPTPN1 were predicted and scored using the online tool SWISS-MODEL based on the comparative homology modelling principle [35]. All the modelling parameters were set to the defaults. Quality assessments of the predicted 3D models, including the Ramachandran plot score and Z score, were performed using the online tools SAVES v6.0 and ProSA-web [36–38]. The 3D structure of UNA was obtained from PubChem (Compound CID: 9890209). The Autodock 4.2 program (genetic algorithm) was used to dock UNA to the active pocket of homoPTPN1, chloPTPN1, and susPTPN1. The estimated free energy of binding was ranked, and the top complex was employed. The docking results were visualized by PyMOL 2.3.2.

### Molecular dynamic simulation

The thermodynamic constancy of the receptor‒ligand system was analysed using Gromacs2021.2 software [39, 40]. First, AmberTools22 was used to add the GAFF force field to the small molecule. Gaussian 16W was used to carry out hydrogenation of small molecules and calculate the RESP potential, and the RESP potential data were added to the molecular dynamics system topology file. Simulations were conducted with the Gromacs package using the Amber99sb-ildn force field at a static temperature of 300 K (the V-rescale method) and 1 bar of atmospheric pressure (the Parrinello-Rahman method). Long-range electrostatic interactions were treated with the particle mesh Ewald method. The Tip3p water model was used to solvate the protein in a periodic dodecahedron box extending 10 Å from the nearest protein atom. The total charge of the simulation system was balanced by adding an appropriate amount of Na^+^, minimized by the steepest descent method, and equilibrated with an isothermal isovolumic ensemble (NVT) and an isothermal isobaric ensemble (NPT) for 100 000 steps, with a coupling constant of 0.1 ps and a duration of 100 ps. All bond lengths were constrained with the LINear Constraint Solver algorithm. A cut-off of 14 Å was used to calculate short-range van der Waals and electrostatic interactions. Finally, a free molecular dynamics simulation was performed. The time step was 2 fs, and the total simulation time was 25 ns. The root-mean-square deviation (RMSD) and the number of hydrogen bonds between the ligand and active pockets were analysed to judge binding stability and convergence.

### Cellular phosphatase activity assay

The cell lysate of Marc-145 cells was generated using cell lysis buffer (Beyotime, China) to measure total phosphatase activity. Subsequently, samples from UNA treatment and the chromogenic substrate para-nitrophenyl phosphate (pNPP, 5 mM) were added to MOPS buffer (20 mM, pH 7.2) in 96-well microplates. The mixture was incubated at 37 °C for 30 min, and the reaction was stopped using NaOH (2 M). The solution absorbance was read at 405 nm.

### Prokaryotic expression of the phosphatase PTPN1

pET-28a-chloPTPN1 was transformed into the *E. coli* strain Rosetta (DE3), and the cells were cultured at 37 °C in LB medium. When the optical density at 600 nm (OD_600_) reached 0.6, isopropyl-β-D-1-thiogalactoside (IPTG, 1 mM) was added to the culture. The cells were harvested after incubation at 37 °C for 6 h, resuspended in PBS and disrupted by ultrasonication. SDS‒PAGE analysis was performed to examine the expression of PTPN1. The recombinant protein precipitate was washed, dissolved in buffer containing 8 M urea, and finally refolded with 0.5 M urea buffer to obtain a refolded protein solution of PTPN1. The above procedures were all performed at 4 °C to avoid unexpected degradation. The protein concentration was determined with a BCA protein quantification kit (Vazyme, China), and the proteins were stored at −80 °C. The purified PTPN1 protein was analysed and verified by SDS‒PAGE and Western blotting with anti-His and anti-PTPN1 antibodies.

### Recombinant PTPN1 activity assay

First, the recombinant phosphatase PTPN1 was prepared in MOPS buffer (20 mM, pH 7.2) at a final concentration of 200 nM. Then, the phosphatase was treated with DMSO or UNA (5, 50, and 500 μM) in MOPS buffer for 30 min. The final volume of each reaction sample was 200 µL. To measure the enzymatic activity of PTPN1, pNPP (5 mM) was added for a 30 min incubation at 37 °C before being stopped with NaOH (2 M). Finally, the solution absorbance in the plate was read at 405 nm.

### Luciferase reporter assay

Marc-145 or HEK-293 T cells were transfected with the IFN-β luciferase reporter plasmid, the pRL-TK Renilla luciferase reporter plasmid (an internal control), the indicated plasmids (wild-type or mutant PTPN1; si-chloPTPN1-1), and the indicated reagents. Then, the cells were stimulated with poly(I:C) (2.5 μg/mL) or RLR-mediated pathway plasmids for 16 h or 24 h. The cell lysates were harvested and subjected to a luciferase assay using a dual-luciferase reporter assay system (Beyotime, China) following the manufacturer’s instructions.

### Broad-spectrum antiviral assessment

RT‒qPCR, Western blotting, and TCID_50_ were used to examine the antiviral effects of UNA on other swine disease viruses. Three designated compound concentrations (2.5, 5, and 10 μM) were added to the culture medium. An equal volume of DMSO was used as the control. PEDV (0.1 MOI) or SVA (0.02 MOI) was then inoculated into Vero or ST cells, respectively. The proteins and supernatants were harvested at 16 h or 18 h post-infection.

### Statistical analysis

All the statistical analyses were performed using GraphPad Prism 7.0 (GraphPad Software, USA), and the data are expressed as the mean ± standard deviation (SD). The significance of differences among groups was determined by one-way or two-way analysis of variance (ANOVA). The asterisks indicate significant differences (*P < 0.05; ** P < 0.01; *** P < 0.001; **** P < 0.0001; ns, not significant).

## Results

### UNA inhibits PRRSV infection in vitro

**Figure 1:**
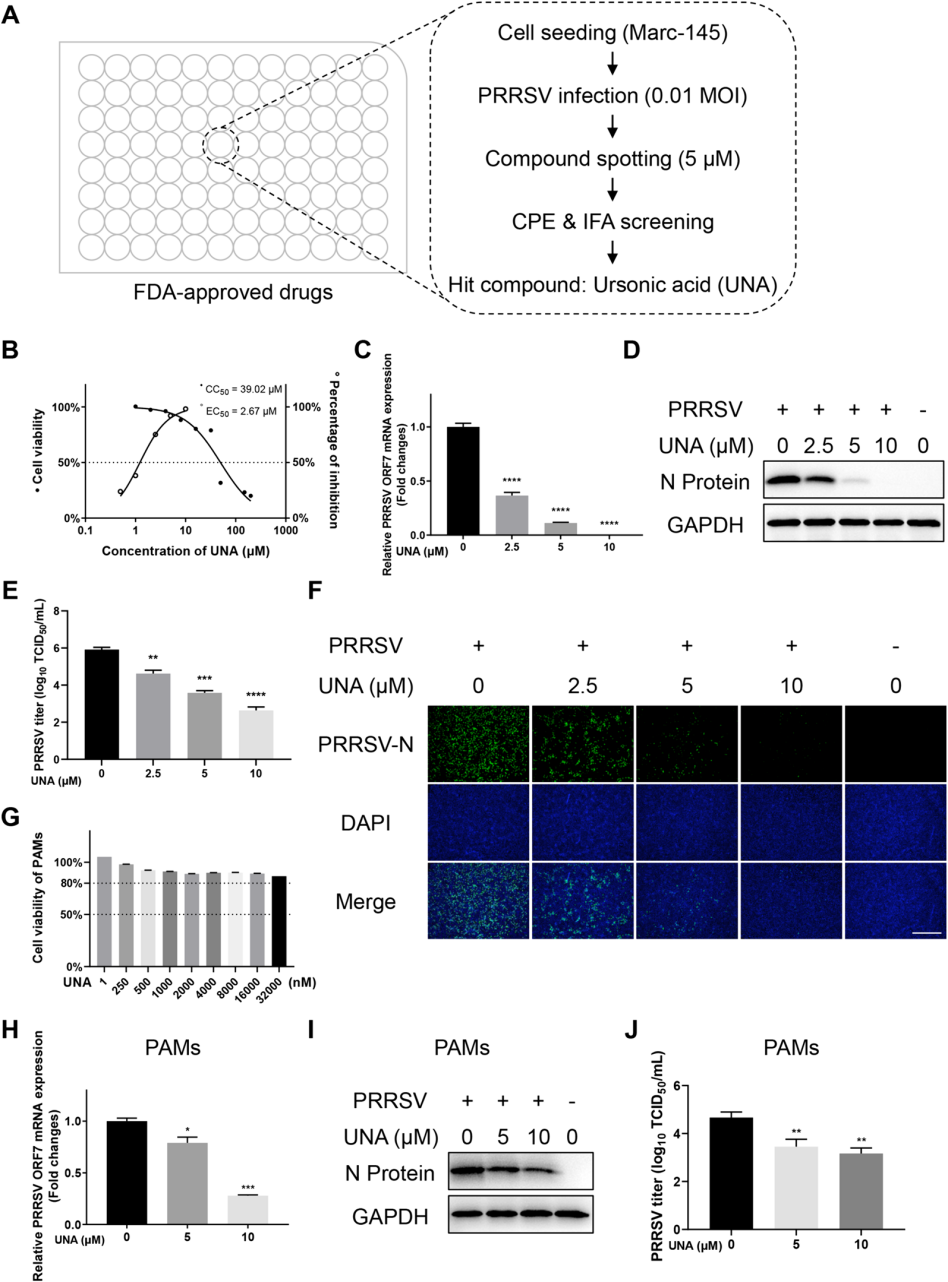
**Identification of the anti-PRRSV activity of ursonic acid (UNA) in vitro.** **A** High-throughput screening (HTS) assay flowchart. **B** 50% cytotoxic concentration (CC_50_) and 50% effective concentration (EC_50_) of UNA in Marc-145 cells. **C** Relative PRRSV ORF7 mRNA levels in Marc-145 cells determined by RT‒qPCR. GAPDH was used as the internal loading control. **D** Western blotting of the PRRSV N protein in Marc-145 cells infected with PRRSV and treated with the indicated concentrations of UNA. **E** Virus titration of samples from Marc-145 cells by TCID_50_ calculation. **F** IFA images of Marc-145 cells (PRRSV-infected and UNA-treated) at 48 hpi. The PRRSV N protein is green, and the nuclei are blue. Scale bars, 500 μm. **G** Viability of PAMs treated with the indicated concentrations of UNA for 24 h. **H** Relative PRRSV ORF7 mRNA levels in PAMs determined by RT‒qPCR. β-Actin was used as the internal loading control. **I** Western blot of the PRRSV N protein in PAMs infected with PRRSV and treated with UNA or DMSO at 24 hpi. **J** Virus titration of samples from PAMs by TCID_50_ calculation. The results are from one of three independent experiments. The data are presented as the means ± SDs. The asterisks in the figures indicate significant differences (*, *P* < 0.05; **, *P* < 0.01; ***, *P* < 0.001; ****, *P* < 0.0001; ns, not significant).

Based on the published results (PMID: 38124181) of a previously conducted high-throughput screening (HTS) assay, the hit compound UNA, which significantly inhibited PRRSV infection at a concentration of 5 μM, was selected for the next step of antiviral efficacy verification (Figure 1A). The cytotoxicity and inhibition rate of UNA on PRRSV were first detected in Marc-145 cells. As shown in Figure 1B, the 50% cytotoxic concentration (CC_50_) and 50% effective concentration (EC_50_) of UNA were 39.02 μM and 2.67 μM, respectively. The selectivity index (SI), determined by the ratio of the CC_50_ to the EC_50_, of UNA reached 14.61. Marc-145 cells were treated with UNA for 1 h before PRRSV infection, and the cells were collected at 48 h post-infection (hpi) for the determination of PRRSV ORF7 mRNA levels, viral N protein expression and the TCID_50_. The results showed that UNA inhibited PRRSV genome replication, viral protein expression and virus production in a dose-dependent manner (Figures 1C−E), which was consistent with the IFA results (Figure 1F).

As the primary target cells of PRRSV infection in pigs, PAMs were treated with noncytotoxic concentrations of UNA (Figure 1G) for 1 h before PRRSV infection to verify the antiviral activity of UNA in porcine cells. RT‒qPCR, Western blot, and TCID_50_ analyses revealed that UNA significantly decreased the PRRSV ORF7 mRNA level, N protein expression, and viral titre in a concentration-dependent manner (Figures 1H‒J). These results demonstrated that UNA effectively inhibited PRRSV infection in vitro.

### UNA disturbs PRRSV infection at different treatment stages

**Figure 2:**
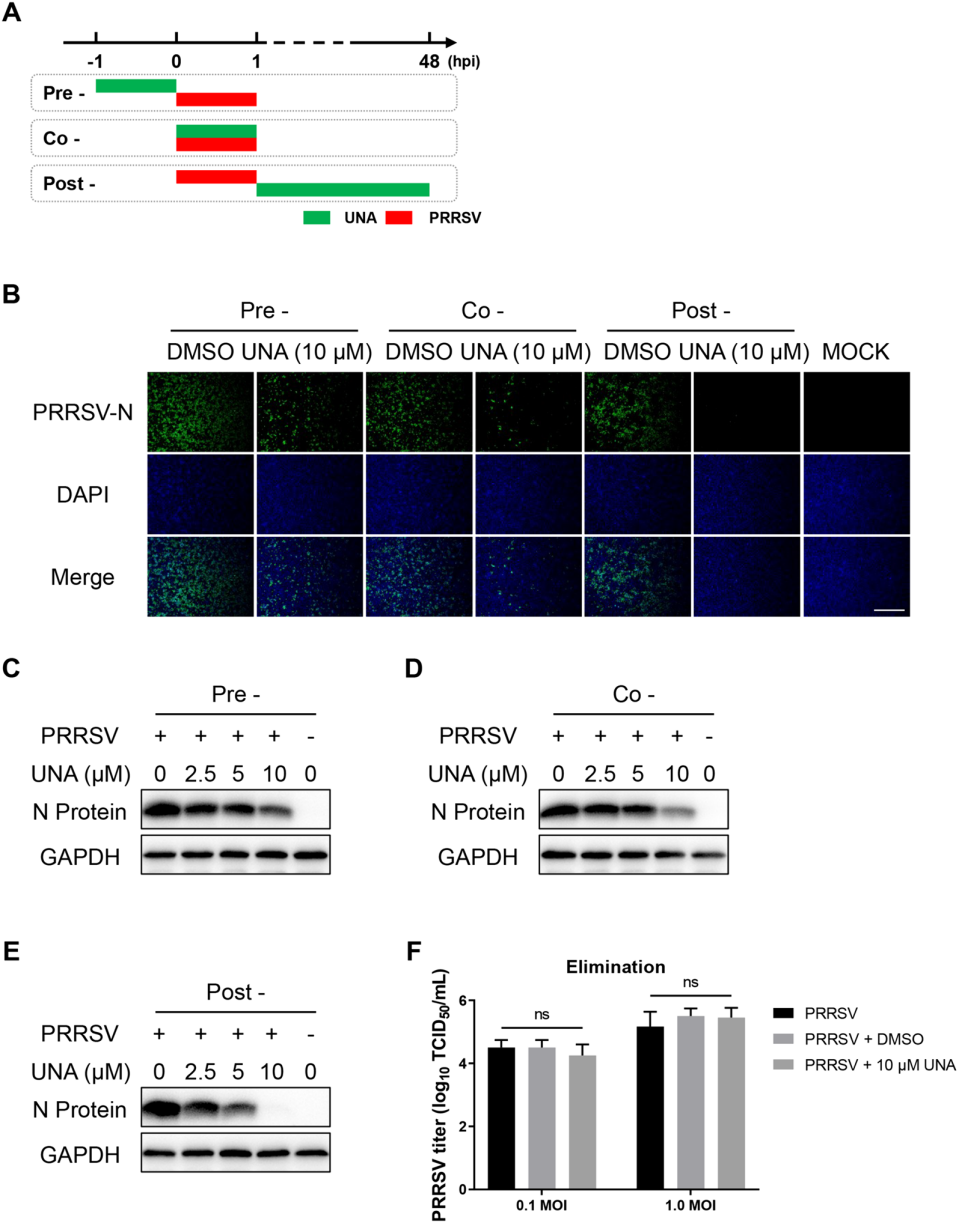
**UNA suppresses PRRSV replication in various treatment stages in the time-of-addition assay. A** Schematic illustration of the time-of-addition experiment. **B** IFA images of Marc-145 cells (PRRSV-infected and UNA-treated) at 48 hpi. The viral N protein is green, and the nuclei are blue. Scale bars, 500 μm. **C**–**E** Western blotting of the PRRSV N protein in Marc-145 cells infected with PRRSV and treated with UNA or DMSO (including pre, co, and post-treatment modes) at 48 hpi. **F** TCID_50_ detection for the virucidal activity assay. The results are from one of three independent experiments. The data are presented as the means** ± **SDs. The asterisks in the figures indicate significant differences (*, *P* < 0.05; **, *P* < 0.01; ***, *P* < 0.001; ****, *P* < 0.0001; ns, not significant).

To explore the mechanism by which UNA inhibits PRRSV, a time-of-addition assay was performed to determine the specific stages at which UNA exerts its antiviral effect (Figure 2A). The IFA results showed that UNA suppressed PRRSV infection in all treatment stages, especially when UNA was administered after PRRSV infection was induced (Figure 2B). Western blot analysis of PRRSV N protein expression showed consistent results (Figures 2C−E). Furthermore, the elimination assay showed that UNA did not have direct virucidal activity against PRRSV (Figure 2F), indicating that the antiviral activity of UNA occurred during virus replication.

### UNA targets and suppresses the phosphatase activity of PTPN1

**Figure 3:**
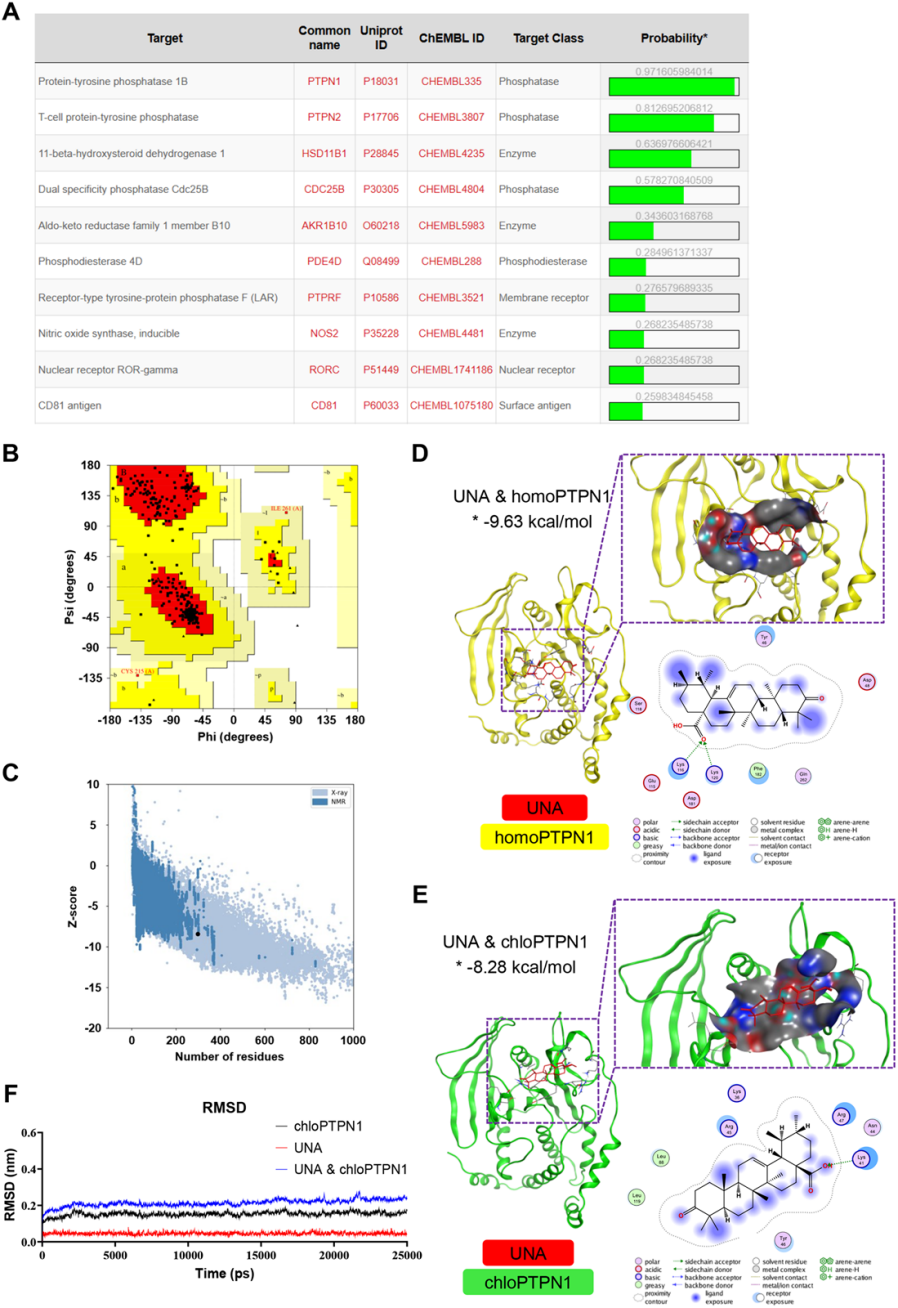
**In silico target prediction and molecular docking analysis of UNA. A** Target prediction for UNA (only the top 10 predicted proteins are listed). **B** The Ramachandran plot statistics of *Chlorocebus sabaeus* PTPN1 (chloPTPN1) represent the most favourable, additional allowed, generously allowed, and disallowed regions, with percentages of 91.1, 8.2, 0.7, and 0%, respectively. **C** The Z score of chloPTPN1 was −8.4. **D** Docked conformation of homoPTPN1 (PDB: 2F71) with UNA. The compound and protein are represented as lines and cartoons, respectively. UNA is coloured red, and the protein homoPTPN1 is coloured yellow. The binding site is shown as a cavity structure. The binding energy of the UNA-homoPTPN1 complex, which was calculated using Autodock, is marked with an asterisk. **E** Docked conformation of chloPTPN1 with UNA. UNA is coloured red, and the protein chloPTPN1 is coloured green. The binding site is shown as a cavity structure. The binding energy of the UNA-chloPTPN1 complex, which was calculated using Autodock, is marked with an asterisk. **F** RMSD values of chloPTPN1 (black), UNA (red), and the UNA and chloPTPN1 complex (blue) over the 25 ns simulation time.

The possible target proteins of UNA were predicted using SwissTargetPrediction (database of *Homo sapiens*) based on its structural similarity to known ligands, of which PTPN1 showed the highest probability of 0.972 (Figure 3A). The structure of *Chlorocebus sabaeus* PTPN1 (chloPTPN1) was predicted by the online tool SWISS-MODEL, and the reliability of the predicted structure was analysed via SAVES v6.0 and ProSA-Web. Ramachandran plot analysis of chloPTPN1 revealed 91.1, 8.2, 0.7, and 0% residues in the most favourable, additional allowed, generously allowed, and disallowed regions, respectively (Figure 3B). The predicted chloPTPN1 had a Z score of −8.4 (Figure 3C).

To further determine whether there is a direct correlation between UNA and PTPN1, molecular docking analysis was conducted via Autodock to assess the possibility of binding between UNA and the enzyme activity site of PTPN1. The estimated binding free energies of UNA & homoPTPN1 and UNA & chloPTPN1 were −9.63 kcal/mol and −8.28 kcal/mol, respectively (Figures 3D and E), indicating that UNA could stably target both homoPTPN1 and chloPTPN1. To analyse the binding stability between UNA and chloPTPN1, a 25 ns molecular dynamic simulation was carried out to obtain the root mean square deviation (RMSD) value. The RMSD can be used to calculate the difference between the observed and estimated values, and a value change of less than 3 Å or 0.3 nm is reasonably acceptable [41, 42]. In the UNA-chloPTPN1 simulation, the RMSD values tended to be stable after approximately 2 ns, and the variation range was lower than 0.1 nm (Figure 3F). These results confirmed that UNA could stably bind to homoPTPN1 and chloPTPN1 in silico.

**Figure 4:**
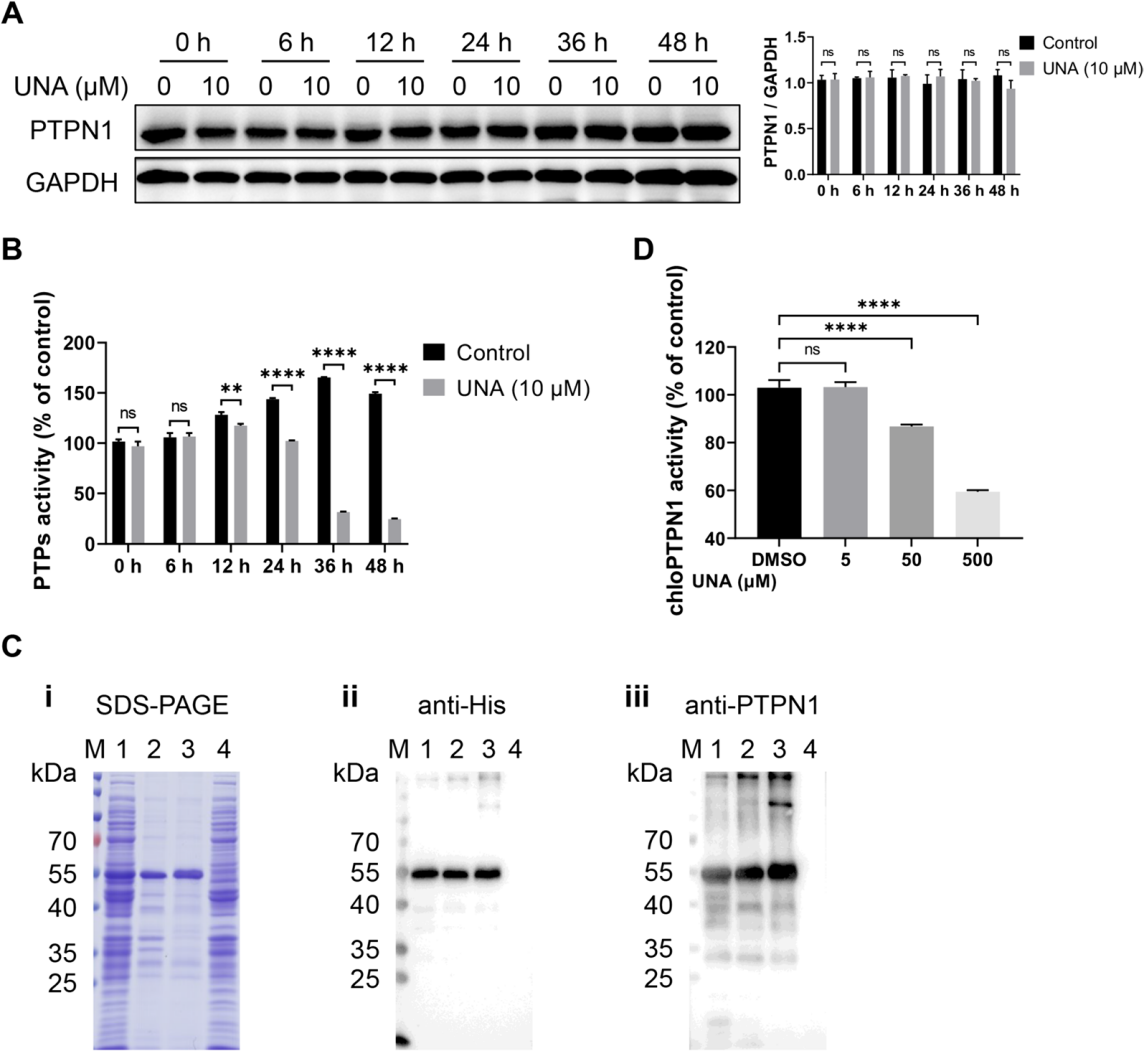
**UNA suppresses the phosphatase activity of PTPN1.** **A** PTPN1 expression in Marc-145 cells treated with DMSO or UNA. The histogram on the right is a statistical analysis of the Western blot data. **B** Protein tyrosine phosphatase (PTP) activity in Marc-145 cells treated with DMSO or UNA. **C** Expression and purification of chloPTPN1. **i** Recombinant protein precipitates before (Lane 2) and after (Lane 3) purification were subjected to SDS‒PAGE. Lane 1, Rosetta-pET-28a-chloPTPN1 with IPTG induction. Lane 4, Rosetta-pET-28a with IPTG induction. **ii** and **iii** The purified protein was analysed by Western blotting. **D** Phosphatase activity of chloPTPN1 after incubation with UNA. The results are from one of three independent experiments. The data are presented as the means ± SDs. The asterisks in the figures indicate significant differences (*, *P* < 0.05; **, *P* < 0.01; ***, *P* < 0.001; ****, *P* < 0.0001; ns, not significant).

As one of the most important class I protein tyrosine phosphatases (PTPs), PTPN1 heavily relies on its phosphatase activity catalytic site ((H/V)C(X)_5_R(S/T)), which catalyses the dephosphorylation of substrates (tyrosine residues in proteins) to exert regulatory functions [19, 43]. The impact of UNA on PTPN1 phosphatase activity was then explored. Marc-145 cells treated with UNA were collected to determine PTPN1 expression and total intracellular phosphatase activity. The results showed that UNA did not affect the expression of PTPN1 (Figure 4A) but significantly inhibited intracellular total phosphatase activity (Figure 4B). To further verify the specific inhibitory effect of UNA on PTPN1 phosphatase activity, chloPTPN1 fused with a His tag was expressed using an *E. coli* expression system. As shown in Figure 4C, panel i, His-tagged PTPN1 was expressed in inclusion bodies. After dissolution, refolding, and purification, the presence of soluble His-tagged PTPN1 was confirmed by Western blotting with an anti-His antibody and an anti-PTPN1 antibody (Figure 4C, panels ii, iii). The results showed that the *E. coli*-expressed PTPN1 protein had high phosphatase activity and that UNA specifically inhibited the phosphatase activity of PTPN1 (Figure 4D). In summary, the above data suggested that UNA targeted PTPN1 and inhibited its phosphatase activity.

### PTPN1 promotes PRRSV replication via its phosphatase activity

**Figure 5:**
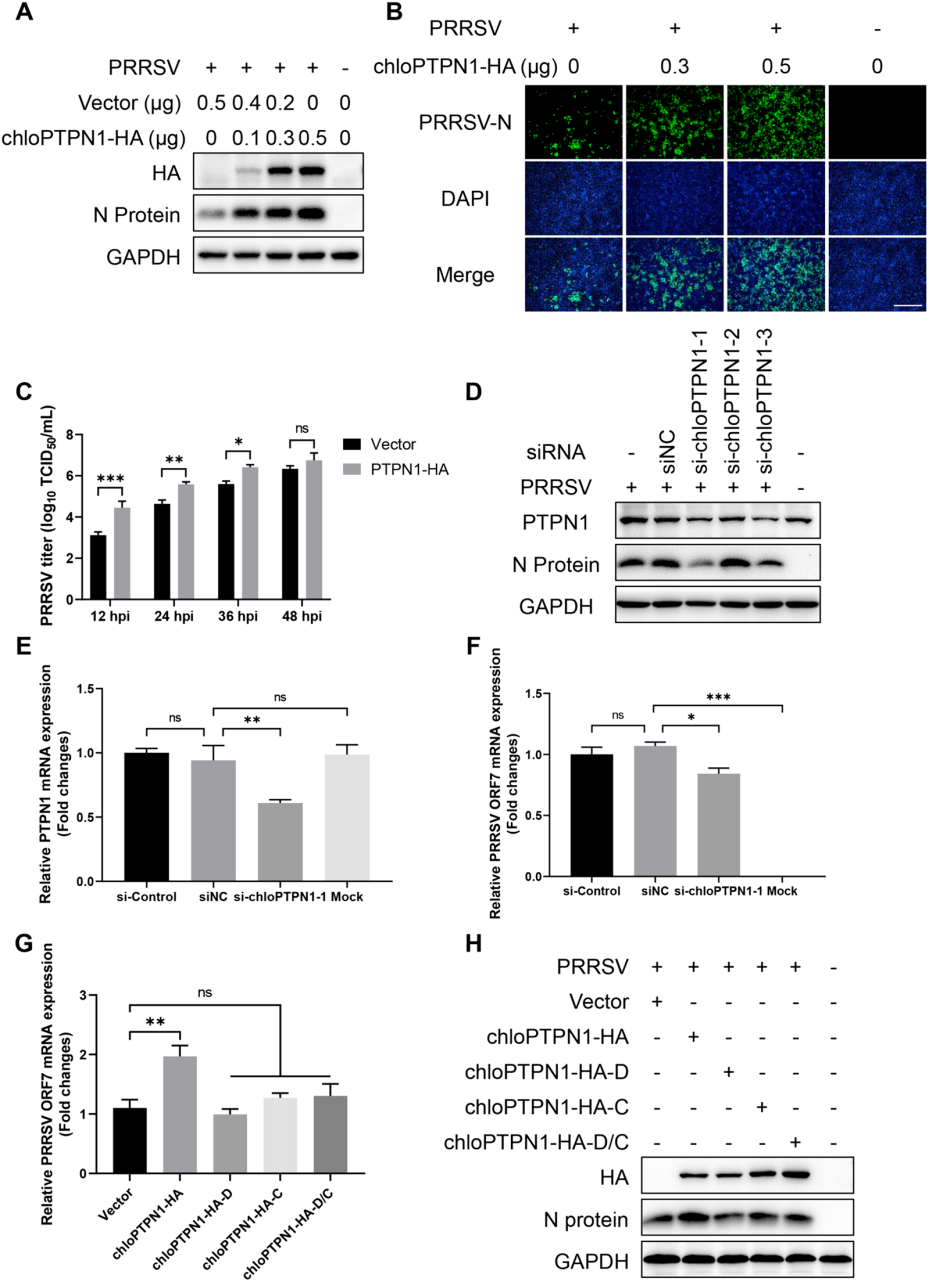
**PTPN1 is a vital proviral factor in PRRSV infection via its phosphatase activity. A** Effect of chloPTPN1 overexpression on PRRSV replication in Marc-145 cells. **B** IFA images of Marc-145 cells (PRRSV-infected and choPTPN1-transfected) at 36 hpi. The viral N protein is green, and the nuclei are blue. Scale bars, 500 μm. **C** Virus titration of samples from Marc-145 cells by TCID_50_ calculation. **D** Western blot analysis of the effect of PTPN1 knockdown on PRRSV replication in Marc-145 cells. **E** and **F** RT‒qPCR analysis of the effect of PTPN1 knockdown on PRRSV replication in Marc-145 cells. **G** and **H** Effects of chloPTPN1 phosphatase activity on PRRSV replication determined by RT‒qPCR (G) and Western blotting (H) in Marc-145 cells. The following plasmids were used: chloPTPN1-HA, chloPTPN1-HA-D181A (“substrate-trapping” mutant, D for short), chloPTPN1-HA-C215S (enzyme-inactive mutant, C for short), and chloPTPN1-HA-D181A/C215S (D/C for short). The results are from one of three independent experiments. The data are presented as the means** ± **SDs. The asterisks in the figures indicate significant differences (*, *P* < 0.05; **, *P* < 0.01; ***, *P* < 0.001; ****, *P* < 0.0001; ns, not significant).

Previous studies have reported that PTPN1 plays vital roles in DNA virus infection and the innate immune response mediated by cGAS/STING or TLRs [24, 44]. The effect of PTPN1 on PRRSV infection was then explored. Marc-145 cells were transfected with pCAGGS-chloPTPN1-HA and then infected with PRRSV. PTPN1 overexpression significantly promoted PRRSV N protein expression (Figure 5A), the proportion of PRRSV-infected cells (Figure 5B), and viral titres from 12 to 36 hpi (Figure 5C). Moreover, the knockdown of PTPN1 by small interfering RNAs (siRNAs) suppressed the viral N protein expression (Figure 5D) and viral genome replication (Figures 5E and F) of PRRSV.

To further examine whether PTPN1 promotes PRRSV infection via its phosphatase activity, a series of chloPTPN1 mutant plasmids were constructed, including the enzyme-inactive mutant chloPTPN1-HA-C215S and the “substrate-trapping” mutant chloPTPN1-HA-D181A and chloPTPN1-HA-D181A/C215S [45]. As shown in Figures 5G and H, only chloPTPN1-HA promoted PRRSV genomic replication and N protein expression, whereas chloPTPN1-HA-D181A, C215S, and D181A/C215S did not. Together, these results demonstrated that PTPN1 is a vital proviral protein for PRRSV infection.

### PTPN1 inhibits poly(I:C)-induced IFN-β production

**Figure 6:**
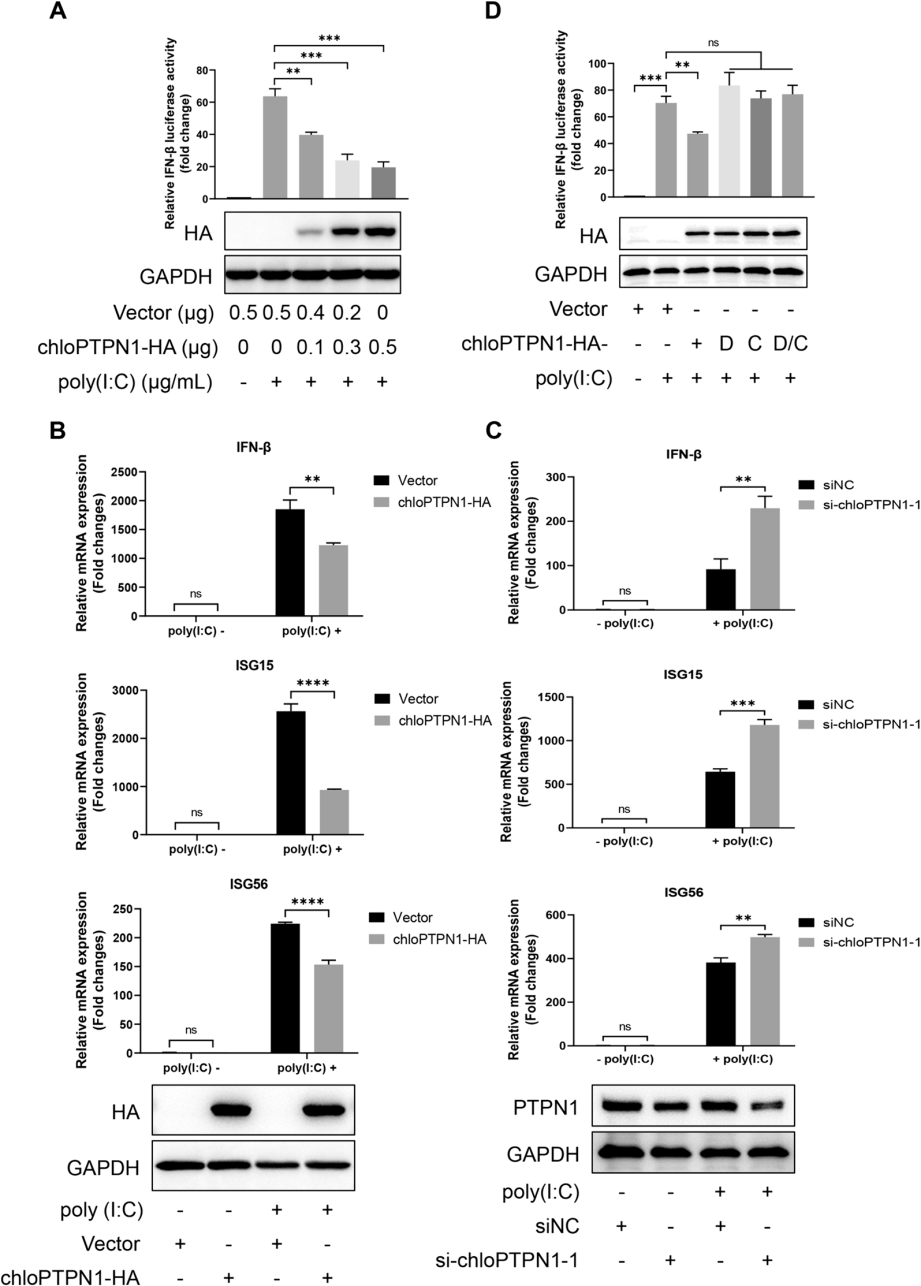
**PTPN1 inhibits the IFN-β promoter stimulated by poly(I:C) via its phosphatase activity.** **A** Luciferase assay and Western blot analysis for assessing the effect of chloPTPN1 overexpression on IFN-β promoter activity in Marc-145 cells. **B** RT‒qPCR and Western blot analysis for assessing the effect of chloPTPN1 overexpression on the mRNA levels of IFN-β, ISG15, and ISG56 in Marc-145 cells. **C** RT‒qPCR and Western blot analysis for assessing the effect of PTPN1 knockdown on the mRNA levels of IFN-β, ISG15, and ISG56 in Marc-145 cells. **D** Effects of chloPTPN1 phosphatase activity on the IFN-β promoter in Marc-145 cells, as determined by luciferase and Western blot assays. The following plasmids were used: chloPTPN1-HA, chloPTPN1-HA-D181A (“substrate-trapping” mutant, D for short), chloPTPN1-HA-C215S (enzyme-inactive mutant, C for short), and chloPTPN1-HA-D181A/C215S (D/C for short). The results are from one of three independent experiments. The data are presented as the means** ± **SDs. The asterisks in the figures indicate significant differences (*, *P* < 0.05; **, *P* < 0.01; ***, *P* < 0.001; ****, *P* < 0.0001; ns, not significant).

Kinase and phosphatase-mediated phosphorylation play essential roles in antiviral signalling pathways [46]. The IFN response is an important pathway for limiting PRRSV infection in hosts [47], and the promotion of PRRSV infection by PTPN1 has been well proven. Therefore, the effect of PTPN1 on the IFN-β response was explored. Dual luciferase assays showed that PTPN1 suppressed poly(I:C)-induced IFN-β production (Figure 6A). Consistently, PTPN1 hindered the poly(I:C)-induced increase in IFN-β, ISG15, and ISG56 mRNA levels (Figure 6B). Conversely, when PTPN1 was knocked down by siRNA, the mRNA levels of IFN-β, ISG15, and ISG56 in poly(I:C)-transduced cells were greater than those in the siNC-transfected cells (Figure 6C). Furthermore, only chloPTPN1-HA inhibited IFN-β promoter activity, while its three mutants that lost phosphatase activity had no effect (Figure 6D), suggesting that PTPN1-mediated suppression of the IFN-β response was dependent on its phosphatase activity.

### PTPN1 attenuates IFN-β production induced by RLR-mediated signalling

**Figure 7:**
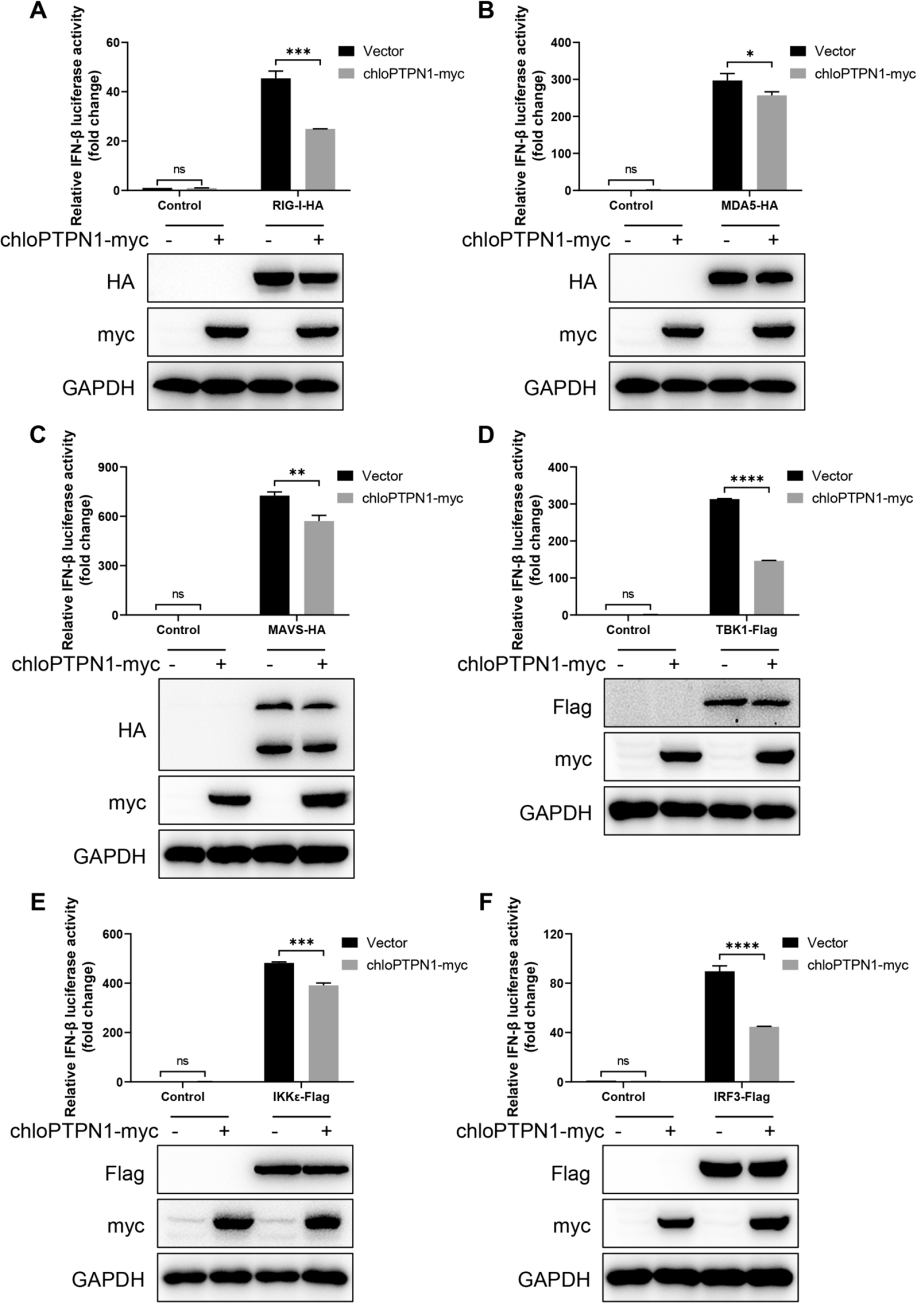
**PTPN1 attenuates IFN-β production induced by RLR-mediated signalling.** HEK293T cells were transfected with the indicated plasmids, chloPTPN1-myc, and the IFN-β luciferase reporter plasmids for 24 h. The cell lysates were harvested for immunoblotting with the indicated antibodies and for luciferase assays to detect the effect of PTPN1 on the activation of the IFN-β promoter induced by RIG-I **A**, MDA5 **B**, MAVS **C**, TBK1 **D**, IKKε **E**, and IRF3 **F**. The results are from one of three independent experiments. The data are presented as the means** ± **SDs. The asterisks in the figures indicate significant differences (*, *P* < 0.05; **, *P* < 0.01; ***, *P* < 0.001; ****, *P* < 0.0001; ns, not significant).

Viral RNAs and poly(I:C) are classical stimulators of IFN-β production via recognition by RIG-I-like receptors [48]. To investigate whether PTPN1 suppressed IFN-β production through the RLR-mediated signalling pathway, the IFN-β luciferase reporter plasmid, the pCAGGS-chloPTPN1-myc plasmid, and the RIG-I, MDA5, MAVS, TBK1, IKKε, or IRF3 expression plasmid were co-transfected into 293 T cells for 24 h. The luciferase results showed that PTPN1 overexpression significantly decreased RIG-I-, MDA5-, MAVS-, TBK1-, IKKε-, and IRF3-induced IFN-β production (Figures 7A−F), implying that PTPN1 might reduce RLR-mediated IFN-β production by affecting IRF3.

### UNA promotes IFN-β production and inhibits PRRSV infection in a PTPN1-dependent manner

**Figure 8:**
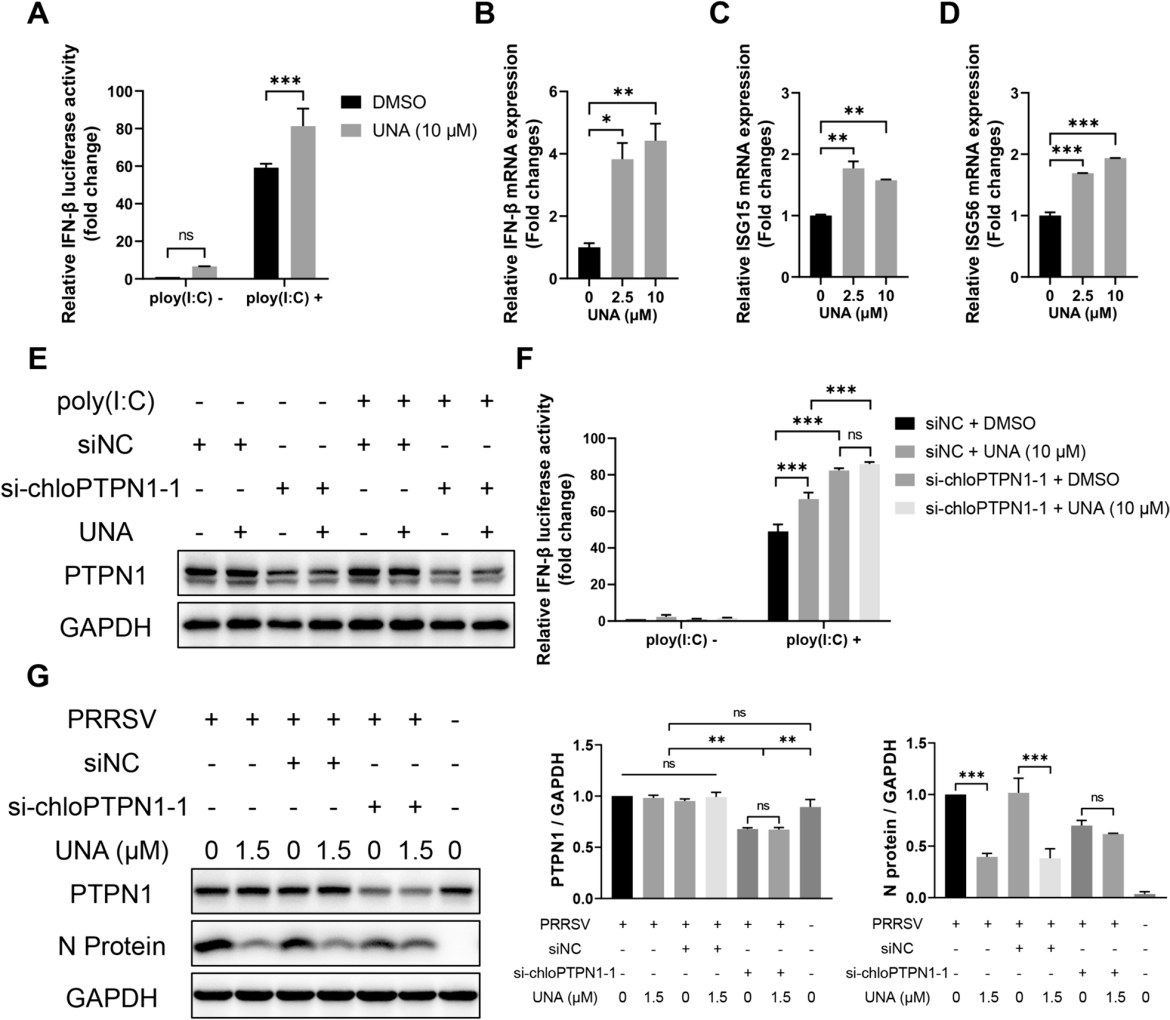
**UNA enhances IFN-β promoter activity and inhibits PRRSV replication via PTPN1.** **A** Luciferase assay for assessing the effect of UNA treatment on IFN-β promoter activity in Marc-145 cells. **B**–**D** RT‒qPCR for assessing the effect of UNA treatment on the mRNA levels of IFN-β, ISG15, and ISG56 in Marc-145 cells. **E** and **F** Luciferase assay and Western blot analysis for assessing the effect of PTPN1 knockdown on UNA-mediated promotion of IFN-β promoter activity in Marc-145 cells. **G** Western blot analysis for assessing the effect of PTPN1 knockdown on UNA anti-PRRSV activity in Marc-145 cells. The histograms on the right show the results of the statistical analyses of the Western blot data. The results are from one of three independent experiments. The data are presented as the means** ± **SDs. The asterisks in the figures indicate significant differences (*, *P* < 0.05; **, *P* < 0.01; ***, *P* < 0.001; ****, *P* < 0.0001; ns, not significant).

To determine whether UNA, a phosphatase activity inhibitor of PTPN1, is involved in the regulation of the IFN-β response, Marc-145 cells were transfected with IFN-β luciferase reporter plasmids and treated with UNA/DMSO for 12 h. Then, poly(I:C) was transfected to stimulate the IFN-β response. The luciferase assay results showed that UNA promoted the activation of the IFN-β promoter (Figure 8A). Consistently, UNA treatment significantly increased the transcript levels of IFN-β, ISG15, and ISG56 mRNA in Marc-145 cells (Figures 8B−D).

To further investigate the role of PTPN1 in the regulation of the IFN-β response and PRRSV infection by UNA, the expression of PTPN1 was silenced by siRNA before UNA treatment for the detection of IFN-β promoter activity and PRRSV N protein expression. As shown in Figures 8E and F, the ability of UNA to increase the activity of the IFN-β promoter was decreased when PTPN1 was knocked down by si-chloPTPN1-1 compared to the siNC control. Consistently, PRRSV N protein expression was decreased in the si-chloPTPN1-1-transfected group, and UNA-mediated inhibition of N protein expression was not detected when PTPN1 was knocked down in Marc-145 cells (Figure 8G). Together, the above-described results demonstrated that UNA exerted promotion of IFN-β production and anti-PRRSV effects through PTPN1, which is consistent with the results of computer simulation analysis and phosphatase activity assays.

### UNA interacts with susPTPN1 and inhibits PRRSV replication in PAMs

**Figure 9:**
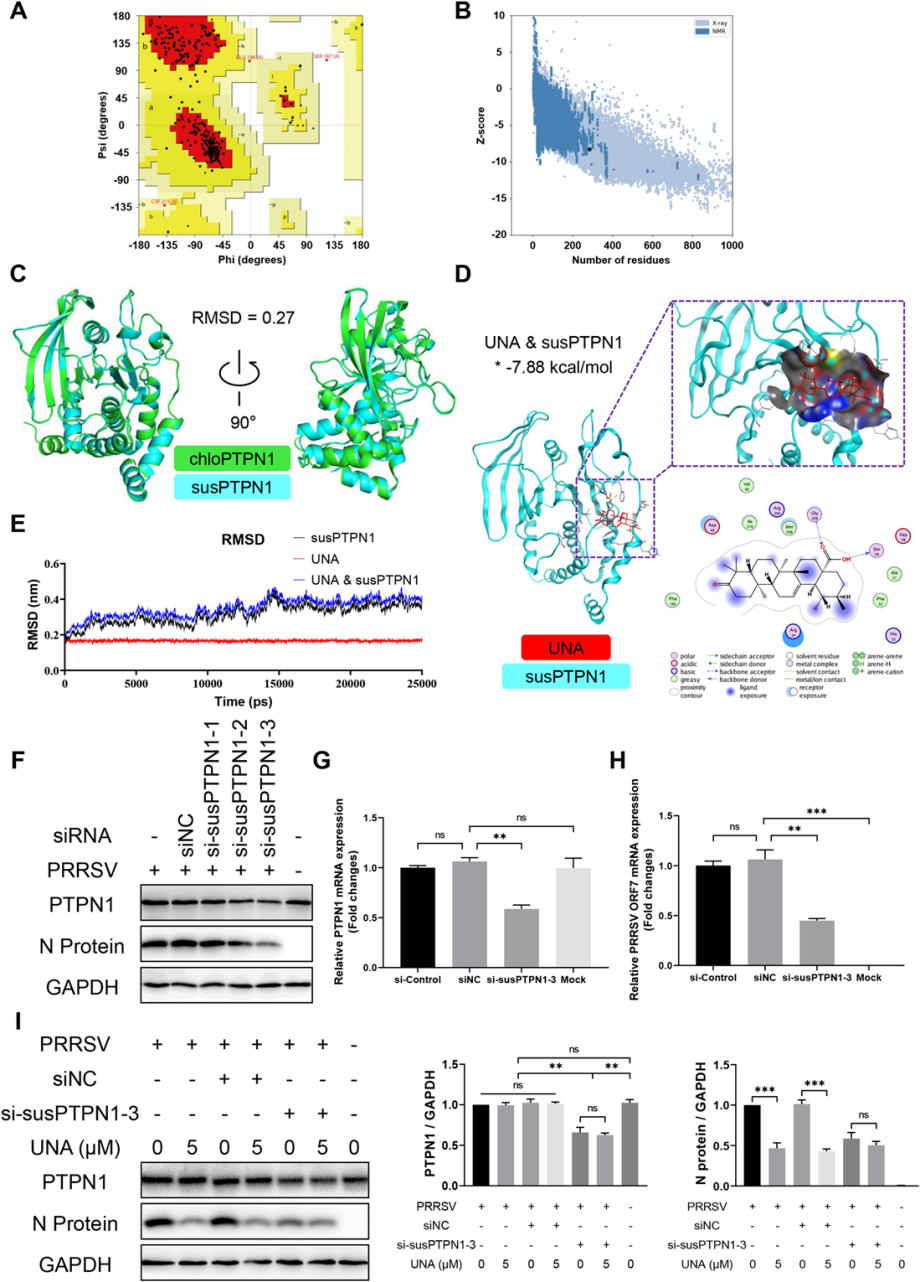
**UNA exerts anti-PRRSV activity by targeting susPTPN1 in PAMs.** **A** The Ramachandran plot statistics of *sus scrofa* PTPN1 (susPTPN1) represent the most favourable, additional allowed, generously allowed, and disallowed regions, with percentages of 88.3, 10.5, 0.8, and 0.4%, respectively. **B** The Z score of susPTPN1 was -8.26. **C** Comparative analysis of chloPTPN1 and susPTPN1 structures by PyMOL. The structures of chloPTPN1 and susPTPN1 are labelled in green and cyan, respectively. **D** Docked conformation of susPTPN1 with UNA. The compound and protein are represented as sticks and cartoons, respectively. UNA is coloured red, and the protein susPTPN1 is coloured cyan. The binding site is shown as a cavity structure. The binding energy of the UNA-susPTPN1 complex, which was calculated using Autodock, is marked with an asterisk. **E** RMSD values of susPTPN1 (black), UNA (red), and the UNA and susPTPN1 complex (blue) over the 25 ns simulation time. **F** Western blot analysis of the effect of PTPN1 knockdown on PRRSV replication in PAMs. **G** and **H** RT‒qPCR analysis of the effect of PTPN1 knockdown on PRRSV replication in PAMs. **I** Western blot analysis for assessing the effect of PTPN1 knockdown on UNA anti-PRRSV activity in PAMs. The histograms on the right show the results of the statistical analyses of the Western blot data. The results are from one of three independent experiments. The data are presented as the means** ± **SDs. The asterisks in the figures indicate significant differences (*, *P* < 0.05; **, *P* < 0.01; ***, *P* < 0.001; ****, *P* < 0.0001; ns, not significant).

The structure of *sus scrofa* PTPN1 (susPTPN1) was predicted and analysed by the online tools SWISS-MODEL, SAVES v6.0, and ProSA-web. Ramachandran plot analysis of susPTPN1 revealed 88.3, 10.5, 0.8, and 0.4% residues in the most favourable, additional allowed, generously allowed, and disallowed regions, respectively (Figure 9A), with a Z score value of -8.26 (Figure 9B). A protein structure alignment between chloPTPN1 and susPTPN1 showed an RMSD of 0.27, which indicated that there was a highly coincident structure between them (Figure 9C). Docking analysis between UNA and susPTPN1 revealed a binding energy of -7.88 kcal/mol (Figure 9D). The binding stability was further analysed by Gromacs2021.2 software through the measurement of RMSD values. The variation range of RMSD values between UNA and susPTPN1 remained lower than 0.3 nm during the 25 ns (Figure 9E), indicating a stable interaction between UNA and susPTPN1.

To determine the effect of PTPN1 on PRRSV infection in PAMs, the cells were transfected with siRNAs targeting susPTPN1. The results showed that the knockdown of susPTPN1 by si-susPTPN1-3 significantly suppressed the viral N protein expression (Figure 9F) and viral genome replication (Figures 9G and H) of PRRSV. When UNA was added to the culture medium, a significant reduction in the PRRSV N protein level occurred only in the noninterference and siNC groups but not in the si-susPTPN1-3 group (Figure 9I), indicating that the anti-PRRSV activity of UNA was achieved by targeting susPTPN1 in PAMs. In summary, the above results suggested that susPTPN1 participated in the replication of PRRSV and that UNA exerted anti-PRRSV activity by targeting susPTPN1 in PAMs.

### UNA shows broad-spectrum antiviral activity against various PRRSV strains and RNA viruses

**Figure 10:**
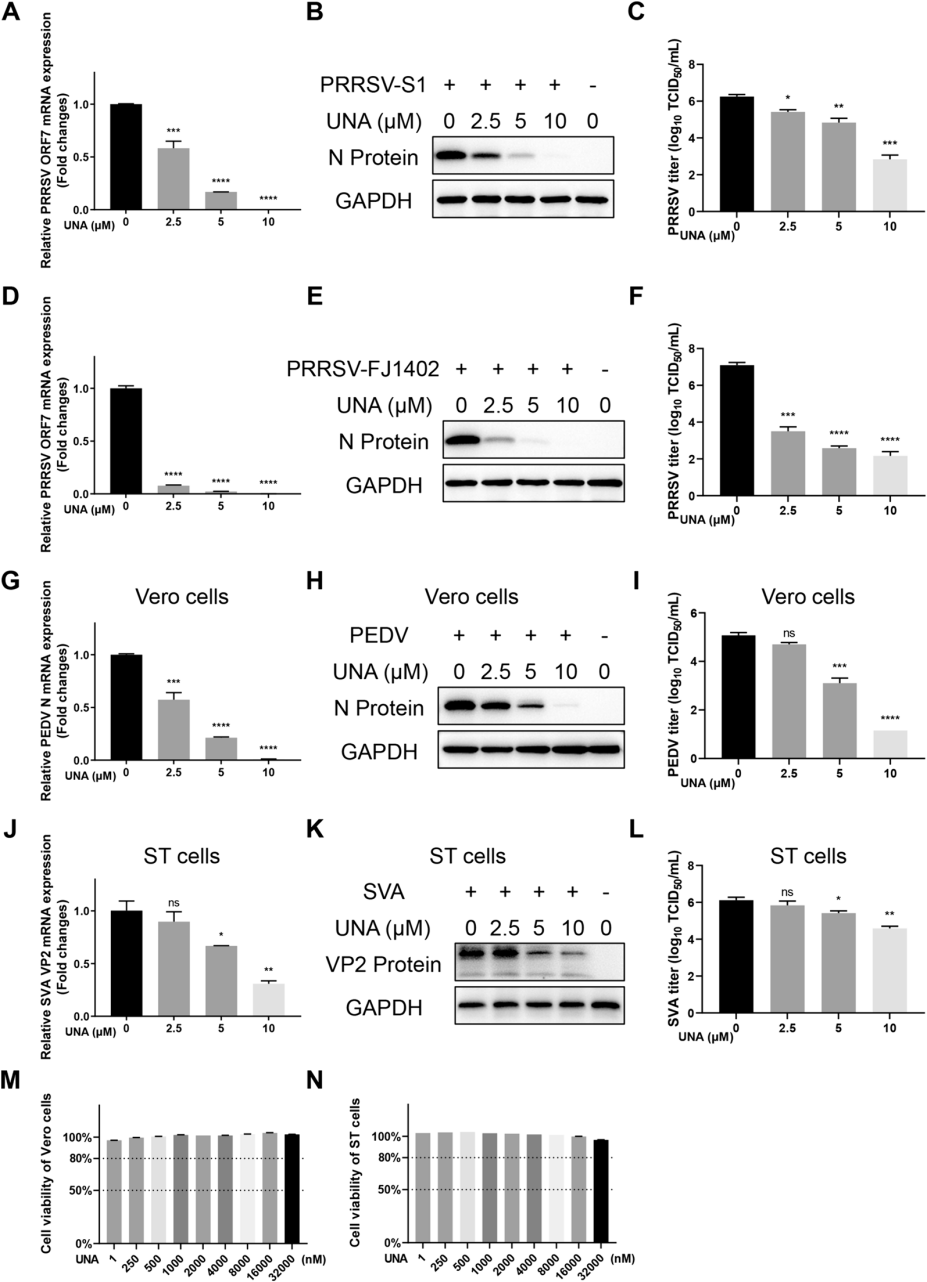
**Broad-spectrum antiviral activity of UNA analysed for various PRRSV strains, porcine epidemic diarrhoea virus (PEDV) and Senecavirus A (SVA).** **A** and **D** Relative PRRSV S1 or PRRSV FJ1402 ORF7 mRNA levels in Marc-145 cells determined by RT‒qPCR. GAPDH was used as the internal loading control. **B** and **E** Western blot analysis of the PRRSV N protein in Marc-145 cells infected with PRRSV S1 or PRRSV FJ1402 and treated with the indicated concentrations of UNA. **C **and **F** Virus titration of samples from Marc-145 cells by TCID_50_ calculation. **G** and **J** The mRNA levels of PEDV N in Vero cells **G** and SVA VP2 in ST cells **J** determined by RT‒qPCR. **H** and **K** Western blot analysis of the PEDV N protein **H** and SVA VP2 protein **K** in Vero or ST cells infected with PEDV or SVA and treated with the indicated concentrations of UNA. **I** and **L** Virus titration of samples from Vero or ST cells for PEDV **I** and SVA **L** detection. **M** and **N** Viability of Vero **M** and ST **N** cells treated with UNA for 24 h. The results are from one of three independent experiments. The data are presented as the means ± SDs. The asterisks in the figures indicate significant differences (*, *P* < 0.05; **, *P* < 0.01; ***, *P* < 0.001; ****, *P* < 0.0001; ns: not significant).

To characterize the antiviral spectrum of UNA, the antiviral effects of UNA against different PRRSV strains (the classic PRRSV S1 strain and the NADC30-like PRRSV FJ1402 strain) were examined using Marc-145 cells via RT‒qPCR, Western blot, and TCID_50_ analyses. As shown in Figures 10A–F, the replication of different strains of PRRSV, reflected by the PRRSV ORF7 mRNA levels, N protein expression, and viral titres, was significantly inhibited by UNA in a dose-dependent manner. These results demonstrated that UNA potently inhibited the viral infection of multiple PRRSV strains.

Previous studies have reported the antiviral effects of UNA on different viruses, such as HSV-1/2, HIV-1, and SARS-CoV-2 [28–30]. To investigate whether UNA has an antiviral effect on other RNA viruses that cause swine diseases, a coronavirus, PEDV, and a picornavirus, SVA, were selected for exploration. The cells were treated with UNA for 1 h and then infected with the indicated viruses for the determination of viral genomic RNA levels, viral protein expression and viral titres. The results revealed that both PEDV and SVA proliferation were significantly suppressed by UNA and were almost completely inhibited at a concentration of 10 μM (Figures 10G−L), and none of the cells treated with the indicated concentrations of UNA exhibited any cytotoxicity (Figures 10M and N). These data indicated that UNA has relatively broad-spectrum therapeutic potential for swine diseases caused by PRRSV, PEDV, and SVA.

## Discussion

Despite extensive and in-depth research on PRRS and its pathogen PRRSV, the control of PRRS remains a major challenge for the global pig industry [49]. The current vaccine-based strategies for the prevention and control of PRRS cannot provide adequate protection for pigs. Thus, novel approaches to control this disease are urgently needed. Natural products and their derivatives have been proven to be important sources for novel antiviral drug development [50, 51]. In this study, we first showed that UNA, a pentacyclic triterpenoid present in various medicinal herbs, dramatically inhibited PRRSV infection at micromolar concentrations in Marc-145 cells and PAMs. UNA has various biological activities, such as antiviral activities against several viruses, as reported in recent years [27, 30, 52]. However, these studies have only reported the antiviral phenotype of UNA without delving into its antiviral mechanism. Here, we elucidated that UNA inhibited PRRSV infection by targeting PTPN1 phosphatase activity, which is the key factor regulating the RLR-mediated IFN-β response.

UNA has a wide range of sources and participates in the regulation of various physiological and pathological processes, including antiviral responses [27]. In 2020, Chen et al. synthesized derivatives of ursolic acid (ULA) and identified their significant antiviral effects on PRRSV in vitro [53]. Although ULA and UNA seem quite similar in chemical structure, they are distinct compounds that exert different biological effects. Our research indicated that UNA could stably inhibit PRRSV infection in vitro.

Phosphorylation, a vital post-translational modification of proteins, plays a crucial role in the regulation of the innate immune response [54]. As one of the essential members of the PTP superfamily, PTPN1 has various cellular functions, such as regulating the innate immune response, which is closely related to viral infections [24]. This study revealed that PTPN1 promotes PRRSV replication via its phosphatase activity. Moreover, PTPN1 is also the key factor in the inhibition of poly(I:C)-induced IFN-β promoter activation. Recognition of the dsRNA analogue poly(I:C) by RIG-I and MDA5 leads to activation of the RLR pathway, which subsequently activates the MAVS-IRF3 cascade and the production of antiviral effector molecules such as IFN-β and interferon-stimulated genes (ISGs) [55]. PTPN1 has previously been shown to be involved in the innate immune response activated by DNA viruses through cGAS/STING [24], but the effect of PTPN1 on RLR-mediated interferon responses has yet to be explored. Our study showed that PTPN1 inhibited RIG-I-, MDA5-, MAVS-, TBK1-, IKKε-, and IRF3-induced IFN-β production, indicating that PTPN1 might target downstream IRF3 to suppress the innate immune response. PTPN1 exerts phosphatase activity by dephosphorylating tyrosine residues of target proteins [19]. For example, PTPN1 negatively regulates insulin metabolism by reducing the phosphorylation level of the insulin receptor [56]. The C-terminal domain of IRF3 contains serine/threonine phosphorylation sites that are critical for its activation and IFN response [57]. In addition, some studies have reported that tyrosine residues, such as Y292 and Y107, are positively regulated in IRF3 during its activation and innate immune response [58, 59]. However, further investigations are needed to determine whether PTPN1 inactivates IRF3 by targeting the tyrosine residues of IRF3.

In this study, the results of bioinformatic analyses and cellular experiments demonstrated that UNA activated the IFN response by targeting and inhibiting PTPN1 phosphatase activity, ultimately inhibiting PRRSV infection. PTPN1 proteins are evolutionarily conserved across different species and share a conserved region in their catalytic site, and the IFN response is a universal pathway for inhibiting viral infections [47, 60]. Therefore, the UNA-PTPN1-IFN response pathway might mediate broad-spectrum antiviral effects, and UNA could be a broad-spectrum antiviral compound. Our study indeed indicated that UNA displayed antiviral efficacy against various RNA viruses that cause pig diseases, such as PRRSV, PEDV, and SVA. Because of the limitations of animal experiments, we did not detect the effect of UNA on the regulation of PRRSV infection in piglets. However, we speculate that UNA has potential as an antiviral drug for the prevention and control of PRRSV since UNA inhibits PRRSV replication in PAMs by interacting with PTPN1, which plays a vital role in regulating the innate immune response. UNA has a wide range of sources with manageable costs, which could make it promising for future applications in the pig industry.

In conclusion, our study revealed that UNA, a natural pentacyclic triterpenoid, is a novel therapeutic agent for combating PRRSV infection. Mechanistic studies revealed that UNA inhibits PRRSV replication by promoting the IFN-β response via the inhibition of PTPN1 phosphatase activity. PTPN1 could be a valuable therapeutic target for drug development against viral infections.

## Data Availability

The data and materials will be made available upon reasonable request.
